# Recent advances in the understanding and management of polycystic ovary syndrome

**DOI:** 10.12688/f1000research.15318.1

**Published:** 2019-04-26

**Authors:** Ana L. Rocha, Flávia R. Oliveira, Rosana C. Azevedo, Virginia A. Silva, Thais M. Peres, Ana L. Candido, Karina B. Gomes, Fernando M. Reis

**Affiliations:** 1 Department of Obstetrics and Gynecology, Universidade Federal de Minas Gerais, Belo Horizonte, Brazil; 2 Department of Internal Medicine, Universidade Federal de Minas Gerais, Belo Horizonte, Brazil; 3 Department of Clinical and Toxicological Analysis, Universidade Federal de Minas Gerais, Belo Horizonte, Brazil

**Keywords:** polycystic ovary syndrome, PCOS, insulin resistance, infertility, menstrual irregularity

## Abstract

Polycystic ovary syndrome (PCOS) is a multifaceted condition characterized by chronic anovulation and excess ovarian activity, in contrast to other causes of anovulation that involve ovarian dormancy or primary insufficiency. Recent studies indicated that PCOS is associated with low-grade chronic inflammation and that women with PCOS are at increased risk of non-alcoholic fatty liver disease. The inflammatory and metabolic derangements associated with PCOS are explained in part by the coexistence of insulin resistance and obesity but are further fueled by the androgen excess. New insights into the regulation of hormones and cytokines in muscle and fat tissue support the concept that PCOS is a systemic syndrome. The therapeutic plan should be tailored to the patient phenotype, complaints, and reproductive desire. Of note, the aromatase inhibitor letrozole seems to be more effective than the reference drug clomiphene citrate to treat infertility due to PCOS. Integral management by a multidisciplinary team may help the patients to adhere to lifestyle interventions and thereby reduce body adiposity and recover their metabolic and reproductive health.

## Introduction

Polycystic ovary syndrome (PCOS) is the most common endocrine disorder in women, presenting with several possible combinations of signs and symptoms and a range of phenotypes, which may include reproductive, endocrine, and metabolic alterations. PCOS is characterized by hypothalamic–pituitary–ovary axis dysfunction and anovulation but, unlike other causes of ovulatory failure that feature insufficient ovarian follicle growth or suppressed gonadotropin secretion (or both), PCOS typically includes androgen excess and subtle alterations (not detected by routine tests) in serum levels of gonadotropins and estrogens. PCOS has the potential for serious consequences, including increased risk for the development of endometrial hyperplasia and neoplasia ^1^. Furthermore, extra-reproductive manifestations of PCOS include insulin resistance (IR), metabolic syndrome (MS), and low-grade chronic inflammation ^2– 6^.

**Figure 1.**
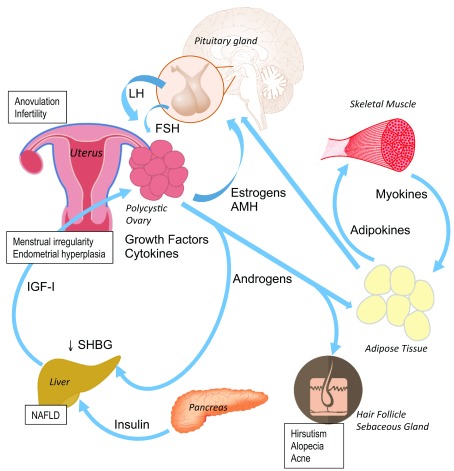
Schematic representation of some pathophysiological mechanisms of polycystic ovary syndrome. The main clinical manifestations are shown in rectangular boxes. Androgens are responsible for dermatological symptoms, while sustained estrogen production by the ovaries and subcutaneous fat without progesterone opposition produces menstrual irregularity and increases the risk of endometrial hyperplasia. Adipokines and myokines may also be involved in the metabolic alterations associated with the syndrome. Insulin resistance and the compensatory hyperinsulinemia are central mechanisms that perpetuate anovulation and lead to metabolic complications. AMH, anti-Müllerian hormone; FSH, follicle-stimulating hormone; IGF-I, insulin-like growth factor I; LH, luteinizing hormone; NAFLD, non-alcoholic fatty liver disease; SHBG, sex hormone–binding globulin.

In recent years, many advances have been made in the understanding of pathophysiological mechanisms ( Figure 1) and thereby in the diagnosis and management of PCOS. We will discuss a limited number of such advancements according to our view of which ones have been more impactful on the health care of women with this syndrome.

## Advances and challenges in PCOS understanding

The Rotterdam Consensus, held jointly by the European and the North American associations of reproductive medicine in 2003, defined the diagnostic criteria of PCOS which remain the most used worldwide for both individual diagnosis and research ^7^. It defined PCOS as the presence of any two of three features: hyperandrogenism (clinical or biochemical), ovulatory dysfunction (often manifested by menstrual irregularities), and polycystic ovarian morphology (PCOM) by ultrasound. The syndrome is a diagnosis of exclusion that should be established only after evaluation of other causes of anovulation or androgen excess (or both): hypogonadism, hypo- or hyperthyroidism, hyperprolactinemia, 21-hydroxylase deficiency, Cushing’s syndrome, and androgen-producing tumors ^7^. Despite some controversy about the appropriateness of the Rotterdam criteria to guide PCOS treatments, their utility to predict reproductive outcomes is well established ^8^. A recent guideline from the International PCOS Network recommended use of the Rotterdam criteria in adults and the requirement of both oligo-anovulation and hyperandrogenism for PCOS diagnosis in adolescents ^9^.

There are several combinations of signs and symptoms that may be accounted for in the diagnosis of PCOS, resulting in different phenotypes for the same syndrome. To better understand the pathogenesis of the syndrome, it is important to compare the genetic profiles of women whose diagnosis was based on the different phenotypes. A recent genome-wide meta-analysis from over 10,000 PCOS cases identified 14 independent loci associated with the risk for PCOS, including three novel loci ^10^. This large-scale study found no difference between the various clinical phenotypes for the association with most of the PCOS-susceptibility loci, suggesting that common genetic traits may underlie the different phenotypes.

Hyperandrogenism is an important clinical characteristic of the syndrome since it is associated with worse prognosis and higher risk of metabolic and cardiovascular disease ^11^. However, recent genetic findings suggest that it may not be the only driver of PCOS manifestations ^10, 12^. As diagnosed by the Rotterdam criteria, hyperandrogenism is present in about 60 to 80% of cases. Biochemical hyperandrogenism remains a diagnostic challenge because the assay methods are poorly standardized, there are no universal cutoffs for diagnosis, and some assays for free testosterone quantification are unreliable ^13, 14^. Thus, until a sensitive, reproducible, and widely validated testosterone assay becomes available and affordable for clinical practice, the assessment of biochemical hyperandrogenism to confirm or discard PCOS should rely on total serum testosterone, sex hormone–binding globulin (SHBG), and free androgen index using local cutoffs ^13, 15^.

Anti-Müllerian hormone (AMH) is a glycoprotein secreted by the granulosa cells of pre-antral and small antral follicles. AMH plays an essential role in sexual differentiation and gonadal function, besides central effects on the hypothalamic–pituitary–gonadal axis. A straightforward experimental study demonstrated that AMH receptor is expressed in gonadotropin-releasing hormone (GnRH) neurons and that intracerebroventricular administration of AMH increases GnRH-dependent luteinizing hormone (LH) pulsatile release ^16^. There is accumulating evidence that GnRH pulsatility is perturbed in women with PCOS, leading to increased LH pulsatility, which plays an important role in PCOS pathophysiology ^17^. Serum AMH levels are typically increased in PCOS ^18^ and therefore AMH-dependent regulation of GnRH release could be involved in the pathophysiology of fertility in women with PCOS ^16^.

### Metabolic profile

IR is so common in PCOS that it can be considered an integral part of the syndrome. IR and glucose metabolism deregulation are currently supposed to play a pathogenic role in the disease. IR leads to compensatory hyperinsulinemia, which increases ovarian androgen synthesis both by direct ovarian actions and by stimulating LH secretion ^3^. IR also induces dyslipidemia, and women with PCOS have an increased risk of type 2 diabetes mellitus and cardiovascular disease ^5^.

Another potential metabolic complication of PCOS is non-alcoholic fatty liver disease (NAFLD), defined as hepatic steatosis not explained by alcohol or other specific etiologic agents. NAFLD is mechanistically and epidemiologically linked to obesity, IR, and MS ^19^. PCOS is associated with increased risk of NAFLD regardless of the presence of obesity, suggesting that the relationship between PCOS and NAFLD is also explained by other features of PCOS, such as IR and androgen excess. In fact, a systematic review and meta-analysis showed a higher prevalence of NAFLD among women with hyperandrogenic PCOS compared with other phenotypes of the syndrome, while serum androgen levels were higher in PCOS women with NAFLD compared with PCOS women without NAFLD ^20^.

Adipokines and inflammation mediators released by the adipose tissue also contribute to the metabolic alterations found in PCOS ^6^. In addition, a number of growth factors, cytokines, and reactive oxygen species produced by the ovaries, liver, and other tissues create a state of chronic inflammation that concurs to maintain the metabolic imbalance of the syndrome ^4, 6^.

During the past decade, skeletal muscle has also been identified as a secretory organ that releases cytokines and other peptides, called myokines. Irisin has been identified as an exercise-induced myokine and has been proposed to mediate the beneficial effects of exercise on metabolism ^21^. Irisin can induce a change in characteristics of white adipocytes that acquire a “brown” phenotype. This change includes the activation of uncoupling protein 1 (UCP-1), leading to increased respiration and energy expenditure ^21, 22^. Irisin is also produced at lower amounts outside the skeletal muscle ^23^. Brain-derived irisin mediates anxiolytic effects of aerobic exercise ^24^ and protects the brain from synaptic and memory loss in an animal model of Alzheimer’s disease ^25^. Furthermore, *in vivo* irisin infusion improves bone mass and architecture in young male mice ^23^.

The expression of irisin is positively associated with body mass index (BMI) and muscle mass, and irisin metabolism is abnormal in patients with type 2 diabetes or gestational diabetes ^26^. Zhang *et al*. observed that serum irisin levels were associated with hyperandrogenism but not with oligovulation or PCOM in women with PCOS ^27^. A recent meta-analysis showed that, after adjustment for BMI, patients with PCOS seem to have normal irisin levels; however, irisin response to hyperinsulinemia might be impaired in patients with PCOS ^28^.

It is uncertain whether PCOS is somehow associated with low serum levels of vitamin D. A systematic review found 12 studies with very heterogeneous results and their meta-analysis suggested lower serum 25-(OH)D in PCOS versus controls but no differences in serum 1,25(OH) _2_D between the two groups ^29^. More importantly, if a woman with PCOS has vitamin D deficiency, she will be at increased risk of metabolic comorbidities. Vitamin D supplementation may reduce chronic inflammation markers in women with PCOS and vitamin D deficiency ^30, 31^ but there is no evidence that such therapy improves the metabolic status of patients ^29^.

### Polycystic ovarian morphology and the diagnosis of PCOS

PCOM is one of three criteria for diagnosis of PCOS. PCOM is defined as follicle number per ovary of at least 12 or ovarian volume of at least 10 mL or both ^7^. However, the latest generation of ultrasound devices (maximal probe frequencies that exceed 8 MHz) is more sensitive and yields a larger follicle count in the general population; therefore, the current use of the old cutoff can overestimate the prevalence of PCOM ^32^. A group of experts recently proposed the use of higher in-house thresholds (that is, 19 to 25 follicles per ovary) to define PCOM with the new ultrasound machines ^33^.

Serum AMH levels correlate with follicle number in women with PCOS. Some studies found a good concordance between serum AMH levels and ultrasound results for the diagnosis of PCOM. These studies suggest that serum AMH could be a proxy for ovarian follicle count and an alternative marker of PCOM to be used interchangeably with ultrasound depending on serum AMH and ovarian follicle count availability ^34^. Recent studies show that high levels of AMH (>35 pmol/L) have a good correlation with the diagnosis of PCOM at ultrasound ^34^. However, serum AMH levels should not yet be used as a marker or as a single test for the diagnosis and detection of PCOS ^9, 33^.

### PCOS at different stages of life

The progression of PCOS during different life stages is poorly known because of the paucity of cohort studies with long-term follow-up. A study compared clinical and biochemical parameters of PCOS women and healthy controls who visited a medical center at a mean age of 29 years and returned 6 years later on average. In this longitudinal sample, aging was associated with an increase in the number of regular menstrual cycles, a decrease in serum androgen levels, and a decrease in IR ^18^. The reasons for this attenuation of PCOS features over time are not clear. Other studies have focused on PCOS manifestations in specific age groups, as detailed below.

***PCOS in childhood.*** The interaction between a genetic predisposition and some prenatal and postnatal environmental factors seems to take part in the pathophysiology of PCOS. Intrauterine growth retardation or small for gestational age (or both) and high levels of androgens during the intrauterine period could lead to an increased production of glucocorticoids which may induce epigenetic modifications and increase the risk of PCOS ^35^.

***PCOS in adolescence.*** PCOS is often diagnosed in adolescence. Menstrual irregularity, acne, and hirsutism are the major findings in this age group. However, these features of PCOS overlap with those of normal adolescence. Family history of PCOS, overweight or low birth weight, exposure to androgens during gestation, precocious puberty, obesity, and IR are risk factors that are related to the development of the syndrome. The diagnosis of PCOS during adolescence is based on stricter criteria than in adult women. It requires unequivocal hyperandrogenism (for example, moderate to severe hirsutism or persistent elevation of serum testosterone levels or both) and ovulatory dysfunction that persists for more than 2 years after menarche ^36^.

Recent studies showed that adolescents with PCOS have increased risk of MS and should be advised to adopt a healthy lifestyle at once ^37^. When a diagnosis of PCOS has been established, the possibility of IR manifestations and quality of life issues should be considered. Obesity, overweight, and hyperinsulinemia may be present in adolescents. In addition, eating disorders (bulimia, anorexia, and binge eating) and inadequate diets with large amounts of hypercaloric and industrialized foods are common in adolescence. Dietary orientation, stimulation to physical activity, and self-care should be part of the integral care for adolescent girls.

***PCOS in postmenopausal women.*** Women with PCOS persist with hyperandrogenism even after menopausal transition and continue to manifest metabolic alterations and MS with increased risk of cardiovascular disease. Therefore, postmenopausal women with a history of PCOS during the reproductive years may still have manifestations of the syndrome ^9, 38^.

## Advances and challenges in PCOS management

Treatment of PCOS should be proposed not only to alleviate symptoms but also to prevent the occurrence of long-term complications. Combined oral contraceptives and antiandrogens are the standard care to reduce androgen levels and treat symptoms while providing endometrial protection ^39^. However, the therapeutic plan should be tailored depending on the desire (or not) of the patient to become pregnant, need for aesthetic approach, and the presence of concomitant metabolic alterations.

**Figure 2.**
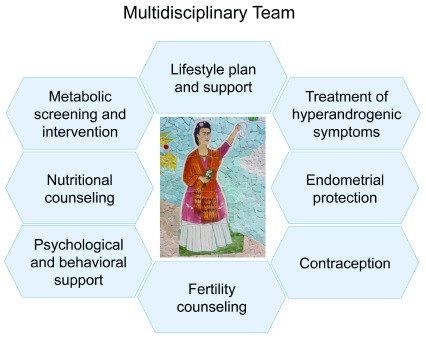
Patient-centered care by a multidisciplinary team may help reach the main goals of polycystic ovary syndrome management. These goals are symptom relief, safe fertility planning, general well-being, and prevention of long-term complications.

The overall goals of therapy of women with PCOS include the mitigation of hyperandrogenic symptoms, management of metabolic abnormalities and reduction of risk factors for type 2 diabetes and cardiovascular disease, prevention of endometrial hyperplasia, planning and obtaining a safe pregnancy if desired, and improving general well-being and quality of life. These goals are ideally achieved by a multidisciplinary team providing patient-centered care ( Figure 2).

### Metabolism

The first line of treatment in patients with PCOS should be the improvement in lifestyle. In overweight and obese patients, weight loss due to changes in diet and physical activity decreases serum insulin and androgen levels and reduces the risk of developing glucose intolerance and type 2 diabetes ^5^. Pharmacological interventions are indicated in the presence of IR/glucose intolerance or dyslipidemia that persist after lifestyle modifications ^40^.

Metformin is the most commonly used drug for the metabolic control of these patients ^41^. The therapeutic effects of metformin as insulin-sensitizing and hypoglycemic agent have been well confirmed in women with PCOS ^42^. However, despite what is commonly believed or what observational uncontrolled studies suggest ^41^, there is no convincing evidence that metformin reduces BMI in women with PCOS compared with placebo ^42^. The addition of metformin may have minimal benefit on the BMI of women who receive antiandrogen and combined oral contraceptive ^39^. Metformin does not seem to decrease body adiposity as it has little if any effect on reducing waist circumference and serum triglyceride levels in women with PCOS ^42^. Current studies are exploring the hypothesis that genomic variants define the responsiveness to metformin therapy among women with PCOS ^43^. Since metformin often has gastrointestinal side effects, new pharmaceutical preparations especially designed for vaginal delivery are under development and so far have been effective in a preclinical model of PCOS ^44^.

Liraglutide is a glucagon-like peptide receptor 1 agonist approved for treating type 2 diabetes and obesity. In obese women with PCOS, liraglutide was effective to induce significant weight loss and reduce waist circumference ^45^. Orlisat is a lipase inhibitor labeled for treatment of obesity. In overweight or obese women with PCOS, orlistat is effective to induce weight loss and improve clinical and biochemical markers of hyperandrogenism and IR ^46^.

Myo- and D-chiro-inositol are insulin-sensitizing agents that act as second messengers in insulin signaling. These compounds have been evaluated as possible alternatives to metformin in PCOS women with IR. Inositol isoforms mediate insulin activity in many target organs, including the ovary, as detailed in a recent review ^47^. In a mouse model of PCOM induced by constant light exposure, the theca/granulosa cell layer thickness ratio and the time to pregnancy were reduced by treatment with myo-inositol and D-chiro-inositol in a 40:1 molar ratio ^48^.

Earlier clinical studies without a placebo or metformin group showed a decrease in serum testosterone along with an increase in SHBG levels after 6 months of treatment with myo-inositol alone or associated with D-chiro-inositol ^49^ and no difference between D-chiro-inositol alone or combined with myo-inositol on the number of mature oocytes retrieved for *in vitro* fertilization (IVF) ^50^. A recent meta-analysis concluded that myo-inositol supplementation for IVF did not improve oocyte or embryo quality ^51^.

Given the current body of evidence, we believe that inositol therapy may become an alternative for metabolic improvement of PCOS women who do not tolerate metformin, but robust data with a face-to-face comparison between inositols and metformin are still missing. Three small, single-center randomized controlled studies published in 2017 addressed this question and found better results with either myo-inositol ^52^ or metformin ^53^ or similar benefits with the two drugs ^54^. According to the International PCOS Network, inositol (in any form) should be considered an experimental therapy in PCOS ^9^.

### Quality of life

PCOS manifests in women at reproductive age when issues such as finding a partner, initiating sex life, and forming a family are often very relevant. Factors that negatively affect physical appearance or femininity or compromise fertility are sources of great anxiety and can lead to imbalances in the psychosexual sphere ^55^. The psychological impact of PCOS may even surpass that of chronic diseases such as asthma, diabetes, arthritis, and coronary heart disease ^56^.

**Figure 3.**
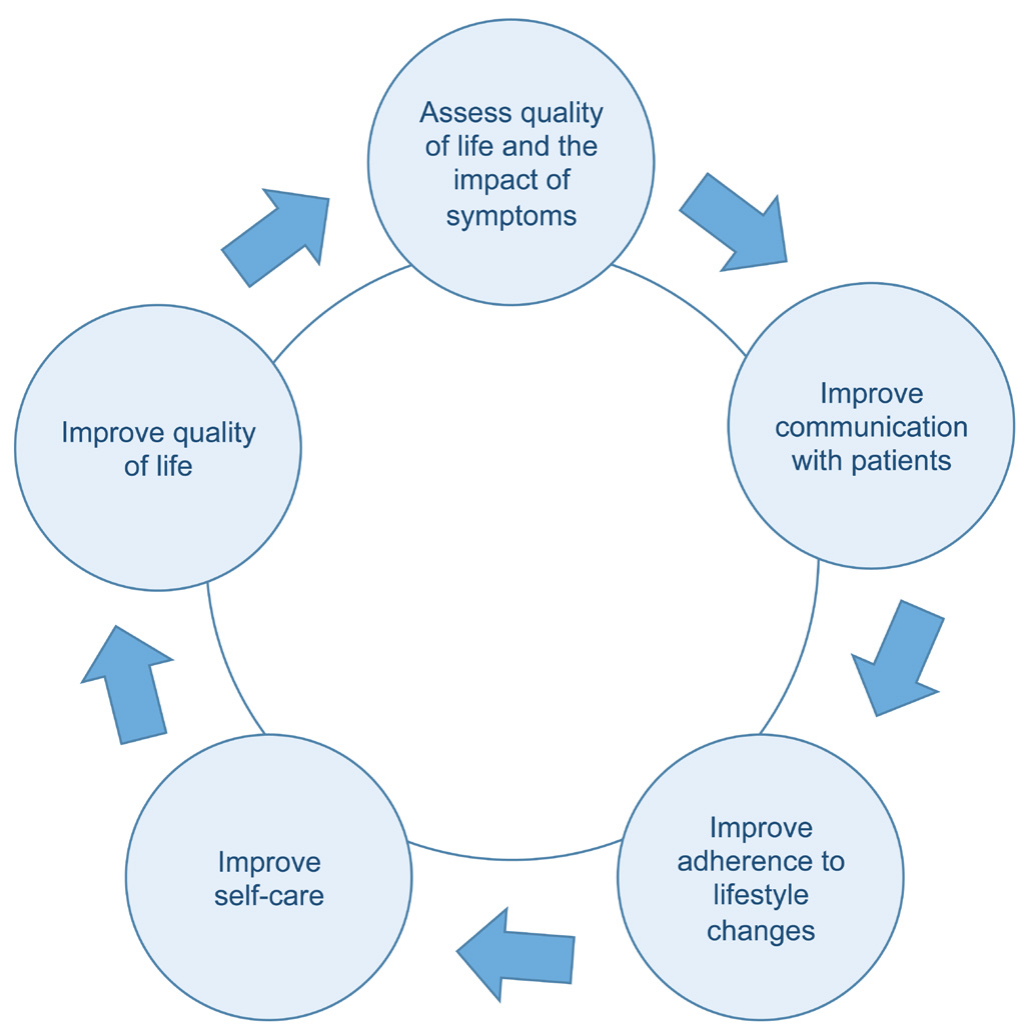
Objectives of assessing the quality of life in women with polycystic ovary syndrome.

Depression and anxiety are highly prevalent in women with PCOS. Dokras *et al*. found a fourfold increase in the prevalence of depressive symptoms in patients with the syndrome when compared with controls, even after adjustment for BMI ^57^. Daily fatigue and sleep disorders, changes in appetite, and loss of interest in everyday activities were the most common symptoms ^58^. Thus, the evaluation of quality of life in women with PCOS is essential for better care and clinical management of these patients ( Figure 3).

### Infertility

In overweight or obese patients with PCOS who wish to conceive, lifestyle modifications aimed at weight loss should be the milestone of preconception counseling. Loss of 5 to 7% of body weight may be effective to promote menstrual cycle regularization and spontaneous ovulation ^40^. If the woman is unable to lose weight or does not restore ovulatory cycles, treatment should be individualized while taking into account the duration of infertility, the woman’s age, the risks of pregnancy at this time, and the factors that may be related to her difficulty to lose weight. Preconception care includes folic acid supplementation at a dose of 0.4 mg/day and cessation of smoking and alcohol consumption.

The second line of treatment (after lifestyle interventions) is ovulation induction. This step must be preceded by careful evaluation of other causes of infertility, such as male factor or tubal obstruction, which demand IVF and may coexist with PCOS.

Clomiphene citrate (CC) is the reference therapy for ovulation induction in anovulatory women with PCOS. In the absence of ovulation for three cycles of CC at the highest dose (150 mg/day), the woman can be considered non-responsive and another drug should be introduced as an adjuvant or substitute for CC.

Compared with placebo, metformin reduces serum testosterone levels and increases the frequency of spontaneous ovulation and regular menstrual cycles in patients with PCOS ^42^. However, because it achieves lower live birth rates compared with CC, metformin is no longer recommended to induce ovulation ^42, 59^. Furthermore, there is no conclusive evidence about whether the association of metformin with CC differs from CC alone in live birth rates. This makes sense, as the addition of metformin to CC increases the ovulation and clinical pregnancy rates but also the miscarriage rate compared with CC alone ^42, 60^. In women resistant to CC, limited evidence suggests that adding metformin may increase the pregnancy rate ^59^. Maintaining metformin during gestation does not seem to prevent adverse outcomes ^42, 59, 61, 62^ and may indeed increase the risk of future overweight in offspring ^63^.

The aromatase inhibitor letrozole at a dose of 2.5 mg/day may be used as an alternative to induce ovulation in patients who have failed to respond to CC. Many studies suggest that letrozole can be used as first-line therapy in ovulation induction but this use remains off-label. The dose may be increased by 2.5 mg/day to a maximum of 7.5 mg/day. The major advantage of letrozole over CC is better ovulation-inducing response, especially in obese patients, which translates into higher pregnancy and live birth rates ^60, 64^. Preliminary data also suggest that letrozole is superior to metformin plus CC to induce ovulation as assessed by the clinical pregnancy rate in an open-label randomized clinical trial ^65^.

In case of failure of oral ovulation inducers to achieve pregnancy, injectable gonadotropins combined with timed intercourse, intrauterine insemination, or IVF may be used. The addition of metformin to gonadotropins has shown some benefit in low-complexity treatments (timed intercourse or intaruterine insemination) but not in IVF ^62, 66^. Preliminary results suggest that liraglutide at low dose can help to improve the outcome of IVF treatment in obese women with PCOS ^67^.

Women with PCOS should be advised about the most opportune moment for pregnancy, taking into account the obstetric, metabolic, and cardiovascular risks that may be present ^68, 69^. Therefore, safe contraception is part of the integral care ^70^ as it allows postponing pregnancy while implementing lifestyle interventions to lose fat mass and improve the metabolic homeostasis in order to obtain not just a pregnancy but a successful full-term pregnancy with both mother and baby in good health.

## Summary and conclusions

New insights into the cross-talk between muscle, fat, brain, and ovary tissue support the concept that PCOS is a systemic syndrome. The classic reproductive and dermatological features of PCOS are just the visible part of a more complex mechanism ( Figure 1). The inflammatory and metabolic derangements associated with PCOS are explained in part by the coexistence of IR and obesity but are further fueled by the androgen excess. The therapeutic plan should be tailored to the patient phenotype, complaints, and reproductive desire. Medical treatments have not seen any breakthrough in recent years. Of note, the aromatase inhibitor letrozole seems to be more effective than the reference drug CC to treat infertility due to PCOS. Integral management by a multidisciplinary team may help patients to adhere to lifestyle interventions and thereby reduce body adiposity and recover their metabolic and reproductive health.
